# Lumican promotes joint fibrosis through TGF‐β signaling

**DOI:** 10.1002/2211-5463.12974

**Published:** 2020-10-25

**Authors:** Dahai Xiao, Tangzhao Liang, Ze Zhuang, Ronghan He, Jianhua Ren, Shihai Jiang, Lei Zhu, Kun Wang, Dehai Shi

**Affiliations:** ^1^ Department of Joint and Trauma Surgery The Third Affiliated Hospital of Sun Yat‐sen University Guangzhou China; ^2^ Department of Plastic Surgery The Third Affiliated Hospital of Sun Yat‐sen University Guangzhou China

**Keywords:** joint capsule synovial fibroblasts, joint contracture, lumican, myofibroblast activation, TGF‐β

## Abstract

Joint contracture (also known as arthrofibrosis) is a fibrotic joint disorder characterized by excessive collagen production to form fibrotic scar tissue and adhesions within joint capsules. This can severely affect day‐to‐day activities and quality of life because of a restricted range of motion in affected joints. The precise pathogenic mechanism underlying joint contractures is not fully understood. Lumican belongs to the class II small leucine‐rich repeat proteoglycan superfamily, which makes up collagen fibrils in the extracellular matrix. Lumican is ubiquitously expressed in the skin, liver, heart, uterus and articular cartilage and has reported roles in cell migration, proliferation, angiogenesis and Toll‐like receptor 4 signaling. Previous research has suggested that lumican is involved in the pathogenesis of several fibrotic diseases. Because joint contracture resembles a fibrotic disease, we aimed to investigate the role of lumican in the development of joint contracture *in vitro*. Here, we showed that protein levels were up‐regulated in the fibrotic joint capsule versus control. We observed that lumican significantly enhanced the proliferation, migration and fibroblast–myofibroblast transition of synovial fibroblasts. Moreover, lumican led to increased transcription of alpha‐smooth muscle actin, matrix metallopeptidase 9, Collagen I, plasminogen activator inhibitor 1 and transforming growth factor‐β *in vitro*. Lumican treatment promoted collagen lattice contraction in a dose‐dependent manner as early as 24 h after treatment. Thus, our studies reveal that lumican could promote fibroblast–myofibroblast transition and joint contracture.

## Graphical abstract

Proposed mechanisms for lumican in joint contracture include: (a) increased lumican with posttraumatic damage that has caused a transition from a normal joint capsule to joint contracture; and (b) stimulating synovial fibroblasts with recombinant lumican leads to increased transcription of alpha‐smooth muscle actin, matrix metallopeptidase 9, Collagen I, plasminogen activator inhibitor 1 and transforming growth factor‐β *in vitro*, possibly through the transforming growth factor‐β pathway. Thus, lumican may affect capsule fibrosis in joint contracture.

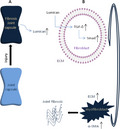

**Abbreviations**
ECM: extracellular matrix
GAPDH: glyceraldehyde‐3‐phosphate dehydrogenase
IHC: immunohistochemistry
MMP‐9: matrix metallopeptidase 9
PAI‐1: plasminogen activator inhibitor 1
PCNA: proliferating cell nuclear antigen
ROM: range of motion
SD: standard deviation
α‐SMA: alpha‐smooth muscle actin
Smad3: mothers against decapentaplegic homolog 3
TGF‐β: transforming growth factor‐β

Joint contracture (also known as arthrofibrosis) is a fibrotic joint disorder characterized by excessive collagen production to form fibrotic scar tissue and adhesions within joint capsules, leading to a limitation in the range of motion (ROM) [1], severely affecting daily living activities [2] and quality of life [3]. Causative factors of joint contracture include arthritis, trauma, chronic or repetitive injuries, or joint surgery [4] that leads to dysregulation of the immune response and fibrosis in a joint [1]. The conventional treatments for joint contracture include rehabilitation and surgery resection of fibrotic tissue; however, only 36% of patients can achieve full recovery, so the efficacy is still unsatisfactory [5, 6]. At present, the precise pathogenic mechanism underlying joint contractures remains not fully understood. Better understanding the pathogenic mechanism may help to develop a novel therapeutic strategy.

Lumican is a member of the class II small leucine‐rich repeat proteoglycans superfamily, which constitutes collagen fibrils in the extracellular matrix (ECM) [7]. Lumican is ubiquitously expressed in a variety of tissues, such as skin [8], liver [9], intervertebral discs [10], kidney [11], bone [12], cornea [7], heart [13], uterus, pancreas [14], intestine, placenta and articular cartilage [15]. Lumican plays a crucial role in the regulation of collagen fibrillogenesis [7] in several tissues, such as skin [8] and cornea [16]. In addition to the functions of collagen fibrillogenesis and water balance in the ECM, lumican has been reported to have important roles in cell migration, cell proliferation [17], wound healing [7], angiogenesis [18], cell migration [19] and Toll‐like receptor 4 signaling [20]. Several lines of evidence suggest that lumican is involved in the pathogenesis of several fibrotic diseases, such as myocardial fibrosis [21], hepatic fibrosis [22], corneal collagen fibrillogenesis [23] and pulmonary fibrosis [24]. Lumican double‐knockout mice have markedly reduced the extent of hepatic fibrosis [22]. Lumican knockout mice exhibit significant corneal posterior opacity, and the lamellar structure was partially disrupted in the posterior stroma with disorganized fibrils [23]. Because joint contracture is also a kind of fibrotic disease, we hypothesized that lumican may have an effect on the pathogenesis of joint contracture.

By using a rabbit model of chronic joint contractures, Hildebrand *et al*. [25] have revealed that the lumican mRNA is up‐regulated in the joint capsules of the contracture knees. This observation suggests that lumican may be implicated in the development of joint contracture. However, the precise role of lumican in the development of joint contracture remains to be investigated. Therefore, the purpose of this study was to elucidate the function of lumican in the development of joint contracture.

## Materials and methods

### Human joint capsule specimens

The joint contracture was one of the complications after elbow replacement. Human fibrosis capsule of elbows were obtained from 18 patients (15 men, 3 women) during operation when we released it. The average age was 50 years [standard deviation (SD), ±4 years; range, 46–54 years] at the time of contracture release, and the release was performed at an average of 12 ± 6 months (range, 6–18 months) after replacement. The average preoperative ROM in the flexion–extension arc was 47°± 23° (range, 24–70°). Thecontrol capsule tissues were collected from 18 normal human knee joint capsules (12 men, 6 women), which were obtained through arthroscopy from the patients’ injured meniscus or anterior cruciate ligament. The tissues were immediately frozen for immunohistochemistry (IHC) analysis using liquid nitrogen. The joint capsule tissue samples were stored at −80 °C until sections were further processed for immunostaining with antibodies. This study was carried out in compliance with the Declaration of Helsinki. All clinical samples were collected in our department, with written informed consent provided by each patient. Ethical approval was granted by the ethical committee of our hospital.

### Cell culture and reagents

Human joint capsule synovial fibroblasts were obtained from the normal knee joint capsule and cultured with Dulbecco's modified Eagle's medium (Nanjing, Jiangsu KeyGEN BioTECH Corp Ltd, China) with 10% FBS (Logan, Utah, Hyclone, USA). Human recombinant lumican protein was purchased from Sino Biological Inc. (Beijing, China) and dissolved in PBS.

### IHC

The sections from joint capsule tissues of the fibrotic joint capsule or normal tissues were immunostained with antibodies against transforming growth factor‐β [TGF‐β; catalog number (cat#): GB11271‐1, 1 : 500 dilution; Wuhan, Servicebio, China], proliferating cell nuclear antigen (PCNA; cat#: GB11010, 1 : 400 dilution;Wuhan, Servicebio), lumican (cat#: ab168348, 1 : 250 dilution; Eugene,Oregon, Abcam, USA) and alpha‐smooth muscle actin (α‐SMA; 1 : 200 dilution; Boston, Massachusetts, Abclonal, USA), as well as Masson trichrome staining to observe the fibrous structure. The sections were visualized by an optical microscope (Eclipse 80i; Nikon Inc., Tokyo, Japan).

The cell nucleus is blue, and the positive result is shades of brown. Finally, quantification of a positive result was achieved with the software imagej (bethesda, maryland, nih, usa）.

### Cell viability assay

Human synovial fibroblasts (5 × 10^3^ cells in 100 μL) were seeded into flat‐bottomed 96‐well plates in 100 mL of growth medium per well and allowed to attach and grow overnight. The medium was then replaced with 100 μL of growth medium containing 0, 10, 50, 100 or 200 nm recombinant lumican and cultured at 37 °C for 12, 24 or 48 h. Untreated cells (0 nm) were used for controls. Cell proliferation viability was assessed by using the Cell Counting Kit‐8 kit according to the manufacturer's protocol (Kumamoto, Kyushu, DOJINDO Laboratories, Japan). The absorbance was measured at 450 nm using a microplate reader (New York 14831, Corning Incorporated, USA). The experiment was repeated three times.

### Cell migration assay

Human synovial fibroblasts (5 × 10^4^ cells) were seeded onto the upper layer of the chamber (24‐well Transwell, 8‐μm pores chamber; Franklin Lake, New Jersey, BD Bioscience, USA) and cultured with 100 µL of the serum‐free medium containing 0, 50, 100 or 200 nm recombinant lumican, whereas 600 µL of complete medium with 10% FBS was added to the lower chamber. After culturing for 24 h, the cells on the side of the chamber were wiped off with a small cotton swab, followed by being fixed with 90% methanol for 10 min and stained with 500 µL of 0.1% crystal violet staining solution at 37 °C for 15 min. The chamber was observed under an inverted Olympus microscope (Tokyo, Olympus, Japan). Five fields of view were randomly selected and photographed, and the number of migrated cells was counted. The experiment was repeated three times.

### Immunofluorescence staining

Human synovial fibroblasts (1 × 10^3^ cells) were cultured in growth medium containing 0, 50, 100 and 200 nm recombinant lumican. After 48‐h incubation, cells were washed with PBS three times. Then cells were fixed in 4% paraformaldehyde for 30 min. The slide was blocked with 100 µL of 20% goat serum for 20 min at room temperature, then by incubation with primary antibodies (1 : 100 dilution) at 37 °C for 2 h, followed by FITC‐conjugated secondary antibody (1 : 100 dilution; Wageningen,KeyGene, the Netherlands) at 37 °C for 1 h in the dark. The slide was then stained with DAPI staining solution at room temperature for 5 min in the dark. The slide was observed by using a fluorescence microscope (Leica MPS30; Wetzlar, Germany). Three fields of view were randomly selected and photographed. Finally, we used the software imagej for quantification of a positive result.

### Quantitative real‐time PCR

Human synovial fibroblasts (2 × 10^5^ cells/well) in six‐well plates were incubated with the serum‐free medium containing 0, 50, 100 or 200 nm recombinant lumican for 48 h. The total RNA was extracted from the fibroblasts using the TRIzol reagent (Carlsbad,California,Invitrogen, USA), according to the manufacturer's protocol as previously described [26]. The first‐strand cDNA was synthesized by using the PrimeScript™ RT Master Mix Kit (Tokyo,Takara, Japan). Quantitative real‐time PCR was performed with the One Step TB Green™ PrimeScript™ RT‐PCR Kit II (SYBR Green) (Takara) on the ABI StepOnePlus real‐time PCR system (Carlsbad, California, Applied Biosystems, USA). Glyceraldehyde‐3‐phosphate dehydrogenase (GAPDH) was used for internal control for normalization. The sequences of the primers used in this study were summarized in Table 1.

**Table 1:** The sequences of the primers used in this study.

	Forward (5ʹ–3ʹ)	Reverse (5ʹ–3ʹ)
Collagen	GAGAGCATGACCGATGGATTC	CTTCTTGAGGTTGCCAGTCTG
MMP‐9	GGCACCACCACAACATCACCTA	CGGGCAAAGGCGTCGTCAAT
PAI‐1	GTTCCAGTCACATTGCCATCA	GGACATTCACTCTGCCACCT
SMAD3	GACCACCAGATGAACCACAGCAT	AGTAGGAGATGGAGCACCAGAAGG
TGF‐β	CTATGACAAGTTCAAGCAGAGT	TGAGGTATCGCCAGGAATTG
GAPDH	AGATCATCAGCAATGCCTCCT	TGAGTCCTTCCACGATACCAA

### Western blot analysis

Western blot was carried out as has been described previously [17]. In brief, human synovial fibroblasts were stimulated with serum‐free medium for 48 h, in the concentrations of 0, 50, 100, or 200 nm recombinant lumican. Total protein extraction from fibroblasts was extracted by using the total protein extraction kit (Jiangsu KeyGEN BioTECH Corp., Ltd) according to the manufacturer's protocol. Protein concentration was determined by the bicinchoninic acid method (Jiangsu KeyGEN BioTECH Corp). The same amount of protein sample was electrophoresed and transferred to the nitrocellulose membrane (Boston, Massachusetts, Millipore, USA). The membrane was blocked with 5% skim milk for 1 h at room temperature and incubated with the primary antibody against TGF‐β (1 : 1000; Jiangsu KeyGEN BioTECH Corp., Ltd.), mothers against decapentaplegic homolog 3 (Smad3; cat#: A16913; Abclonal), plasminogen activator inhibitor‐1 (PAI‐1; cat#: A6211; Abclonal), Collagen I (cat#: ab138492; Abcam), matrix metallopeptidase 9 (MMP‐9; Abclonal) and GAPDH (cat#: A0289; Abclonal) at 4 °C overnight. After washing, the membrane was incubated with the goat anti‐rabbit IgG‐horseradish peroxidase secondary antibodies (Jiangsu KeyGEN BioTECH Corp.) at room temperature for 1 h. The membranes were developed with the chemiluminescent substrate kit (Jiangsu KeyGEN BioTECH Corp) and visualized using a G‐BOX ChemiXR5 imaging system (Syngene, Cambridge, UK).

### Collagen lattice contraction assay

Human synovial fibroblasts were cultured with serum‐free medium for 24 h. Rat‐tail collagen gel was diluted four times with incomplete medium, and 1 mL of diluted rat tail collagen gel (250 μL of rat tail collagen gel; 20 μL of 0.1 N NaOH; 630 μL of RPMI 1640; 100 μL of FBS) was added to a well of the 12‐well plate and incubated at 37 °C for 60 min to polymerize the gel to form the collagen lattice. Synovial fibroblasts (1 × 10^4^ cells in 100 µL serum‐free medium) without or with recombinant lumican at final concentrations of 0, 50, 100 or 200 nm were added onto a well of collagen lattice, followed by incubation in 37 °C incubator for 12, 24 or 48 h. The extent of collagen matrix contraction was then photographed.

### Statistical analysis

Data were represented as the mean ± SEM in three separate experiments performed in triplicate. The Student's *t*‐test was conducted when comparing two groups, and one‐way ANOVA was conducted when comparing more than two groups, using graphpad prism 7.0 software (GraphPad Software, Inc., La Jolla, CA, USA). *P* < 0.05 was considered to indicate a statistically significant difference.

## Results

### Lumican protein was up‐regulated in the fibrotic joint capsule

**Fig. 1:**
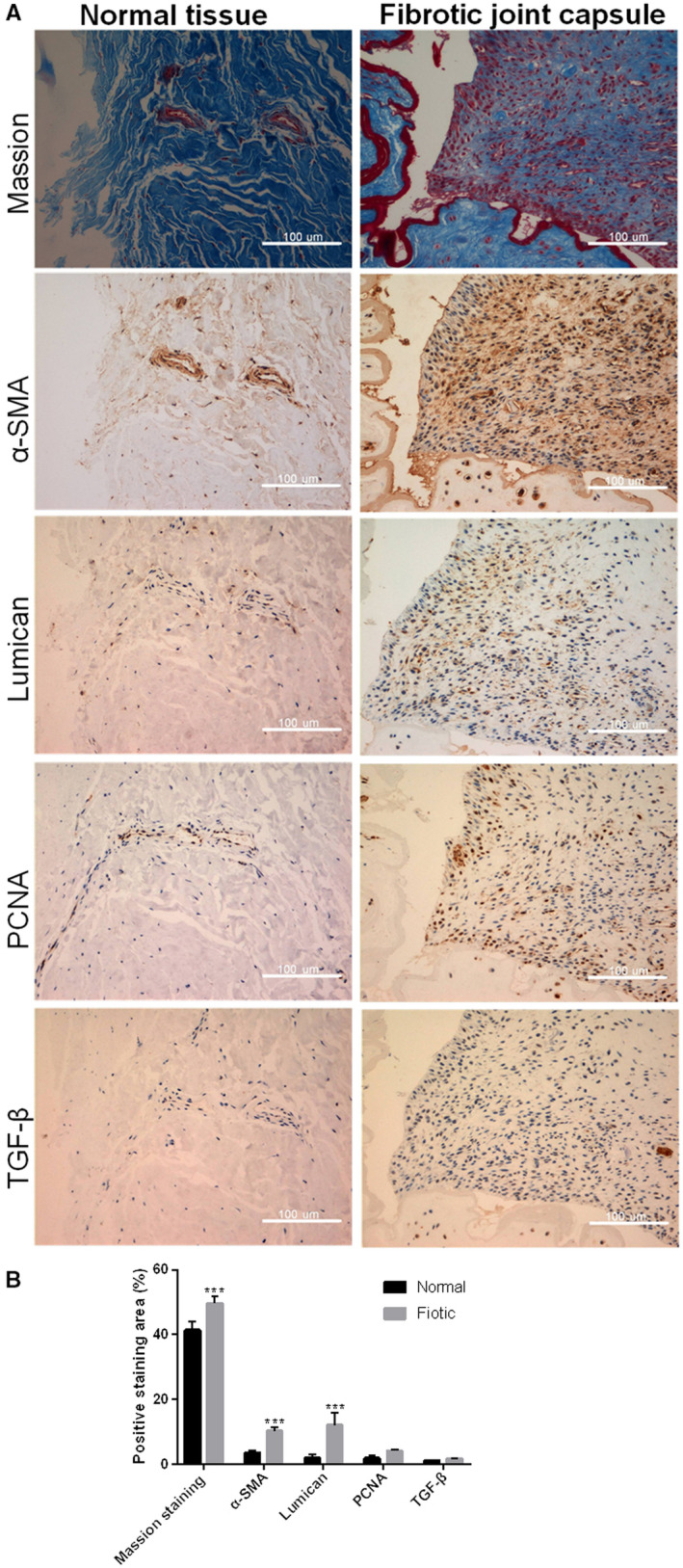
The expression levels of α‐SMA, lumican, PCNA and TGF‐β *in vivo*. (A) Masson staining and IHC analysis for α‐SMA, lumican, PCNA and TGF‐β in the joint capsule tissues from the fibrotic joint capsule and normal tissues. Scale bars: 100 μm. (B) Bar chart showed quantitative results of the Masson staining (Collagen) and IHC analysis. Data were presented as mean ± SD for three independent experiments. The Student's *t*‐test was conducted. ***P < 0.001, compared with the normal groups.

To investigate whether lumican is involved in the development of joint contracture, we compared the protein level of lumican between human contracture joint capsule with fibrotic contracture and normal tissues by IHC analysis (Fig. 1A). The results showed that lumican was significantly up‐regulated in the fibrotic joint capsule as compared with normal tissues (Fig. 1B, *P* < 0.001). Meanwhile, the protein level of α‐SMA was also significantly up‐regulated in the fibrotic contracture tissues compared with the normal tissues (Fig. 1A,B, *P* < 0.001). Finally, the protein level of PCNA and TGF‐β were also increased in the fibrotic contracture tissue compared with control, but this does not indicate a statistically significant difference.

### Exogenous lumican treatment promoted proliferation and migration of human joint capsule synovial fibroblasts

**Fig. 2:**
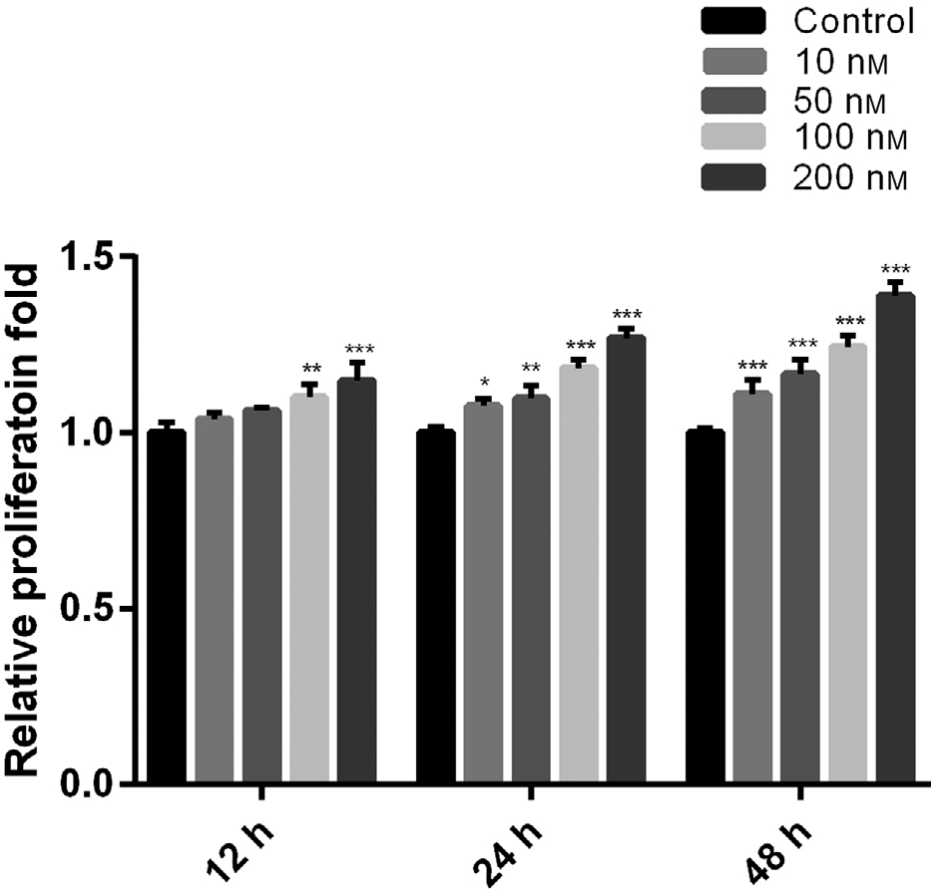
Lumican promotes the proliferation of fibroblasts. Synovial fibroblasts were treated with lumican (0, 10, 50, 100 and 200 nm) for 12, 24 or 48 h. Data were presented as mean ± SD for three independent experiments. The Student's *t*‐test was conducted. *P < 0.05; **P < 0.01; ***P < 0.001, compared with the control group.

Because lumican was up‐regulated in fibrotic contracture tissue, we addressed whether lumican has an effect on the biological activity of human synovial fibroblasts. Cell Counting Kit‐8 assay demonstrated that compared with the untreated control, exogenous lumican treatment (100 and 200 nm) significantly enhanced proliferation of synovial fibroblasts at 12 h (both *P* < 0.01; Fig. 2). At 24 and 48 h, the proliferation of synovial fibroblasts was significantly up‐regulated in all of the lumican treatment groups (10, 50, 100 and 200 nm) in a dose‐dependent manner as compared with the control group (all *P* < 0.05; Fig. 2).

**Fig. 3:**
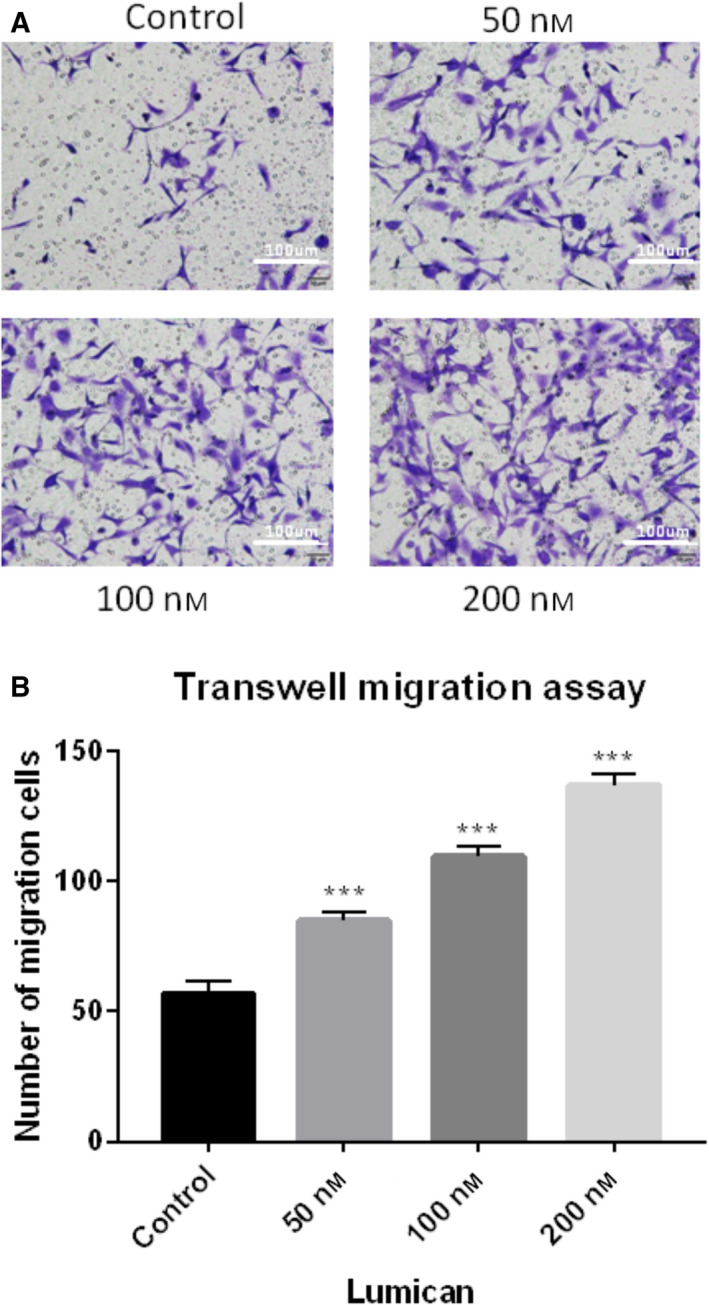
Lumican promotes the migration of fibroblast. (A) Transwell assay showed the myofibroblast migration ability in different groups. Scale bars: 100 μm. (B) Bar chart showed the quantitative results. Data were presented as mean ± SD for three independent experiments. The Student’s *t*‐test was conducted. ***P < 0.001, compared with the control group.

Cell migration was assessed by Transwell assay. As shown in Fig. 3, lumican treatment significantly promoted cell migration of synovial fibroblasts in a dose‐dependent manner (all *P* < 0.001).

### Lumican promoted fibroblast transition to myofibroblast

**Fig. 4:**
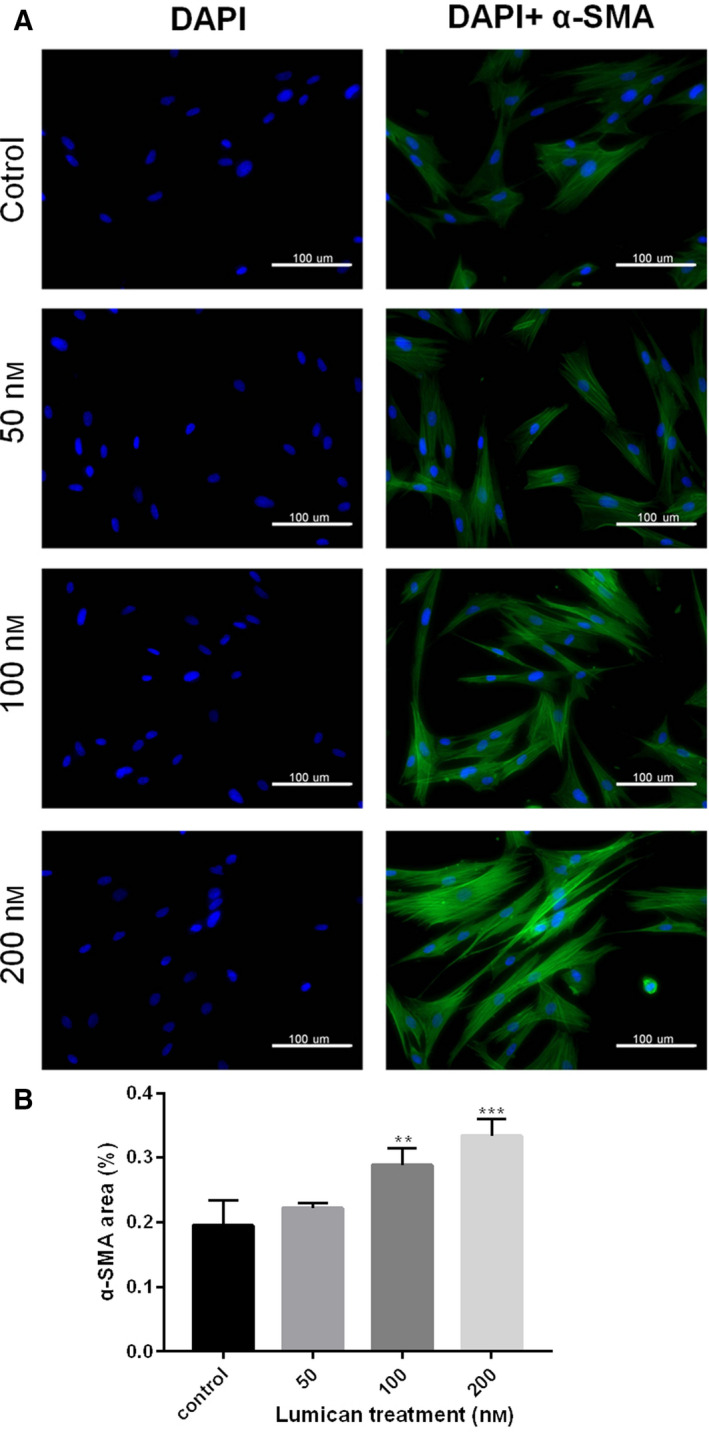
Lumican promotes the activation of fibroblasts. (A) Cell nuclei were stained with DAPI (left panels). α‐SMA proteins were stained as green fluorescence (right panels). The right panels showed the merged image of DAPI and α‐SMA staining. Scale bars: 100 μm. (B) The quantitative result of immunofluorescence staining for α‐SMA was shown in the bar chart. Data were presented as mean ± SD for three independent experiments. The Student's *t*‐test was conducted. **P < 0.001; ***P < 0.001, compared with the normal group.

Fibroblast transition to myofibroblast is a key event in the onset of fibrosis, and myofibroblasts are responsible for producing ECM proteins (such as collagen) in the fibroproliferative diseases [27]. To investigate whether lumican affects fibroblast–myofibroblast transition, we performed immunofluorescence to assess the protein level of α‐SMA (a myofibroblast marker). The results revealed that lumican treatment significantly up‐regulated α‐SMA proteins in a dose‐dependent manner (Fig. 4A,B), suggesting that lumican treatment enhanced fibroblast–myofibroblast transition.

### Lumican treatment increased the expression of TGF‐β, MMP‐9, collagen and PAI‐1

**Fig. 5:**
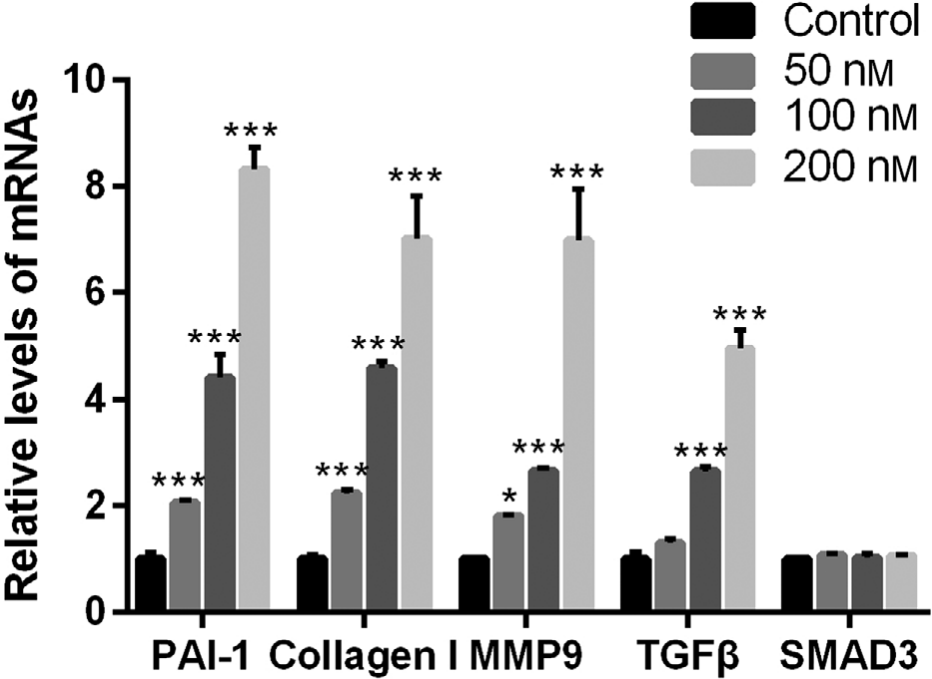
Lumican promotes the expression of mRNA in fibrosis. The chart showed the expression levels of PAI‐1, collagen, MMP‐9, TGF‐β and Smad3 with human synovial fibroblasts treatment with lumican (0, 50, 100 or 200 nm) for 48 h. Data were presented as mean ± SD for three independent experiments. The Student's *t*‐test was conducted. *P < 0.05; ***P < 0.001, compared with the control group.

**Fig. 6:**
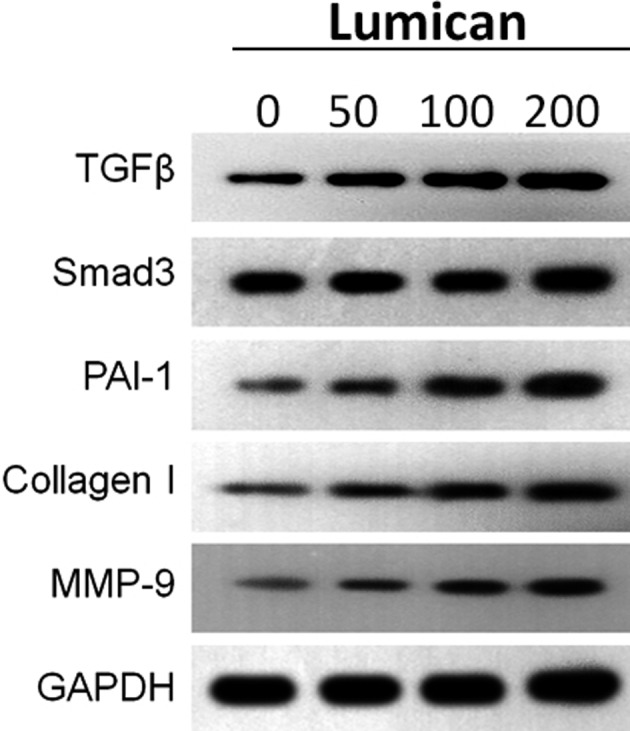
Lumican promotes the expression of protein level in fibrosis. The protein expression levels of TGF‐β, SMAD‐3, PAI‐1, Collagen I, MMP‐9 and GAPDH of human synovial fibroblasts treatment with lumican (0, 50, 100 or 200 nm) for 48 h. The experiment was repeated three times.

Next, we attempted to address the molecular mechanism underlying the promotive effect of lumican on fibroblast–myofibroblast transition. Real‐time RT‐PCR showed that lumican treatment significantly up‐regulated mRNA expressions of TGF‐β, MMP‐9, collagen and PAI‐1 in synovial fibroblasts in a dose‐dependent manner (all *P* < 0.001; Fig. 5). However, the level of SMAD3 was not affected by lumican treatment (*P* > 0.05; Fig. 5). In addition, western blot analysis consistently demonstrated that lumican treatment considerably up‐regulated protein levels of TGF‐β, MMP‐9, collagen and PAI‐1, but not SMAD3 (Fig. 6). These results suggested that lumican may enhance fibroblast–myofibroblast transition via TGF‐β signaling.

### **Lumican treatment promoted collagen contraction** *in vitro*

**Fig. 7:**
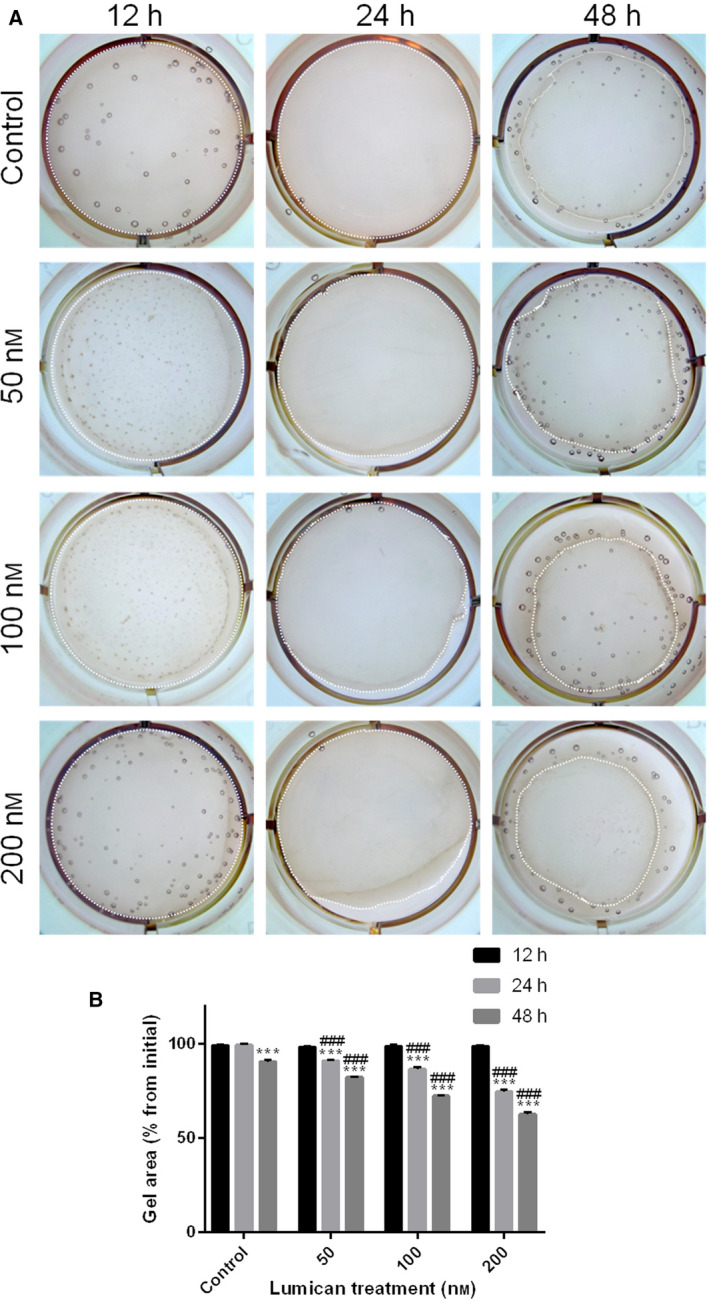
Lumican promotes the contractile ability of human synovial fibroblast in 3D collagen gels. (A) Representative images of collagen gel contraction after treatment with lumican (0, 50, 100 or 200 nm) for 12, 24 or 48 h. The white spots outline the gels. (B) The gel area was quantitatively determined and presented as percentage of initial area. Data were presented as mean ± SD for three independent experiments. The Student's *t*‐test was conducted when comparing two groups, and one‐way ANOVA was conducted when comparing more than two groups. ***P < 0.001, compared with the 12 h within the same group; ^###^ *P* < 0.001, compared with the control group with the same time point.

Because lumican treatment enhanced fibroblast–myofibroblast transition, we investigated whether lumican directly induces tissue contraction. To this end, the collagen lattice contraction assay was performed with different concentrations of lumican (50, 100 and 200 nm; Fig. 7A). It was found that lumican treatment (50, 100 and 200 nm) started to induce significantly collagen lattice contraction at 24 h and became more significant at 48 h as compared with the control group (Fig. 7B, all *P* < 0.001). Moreover, in comparisons among the 50, 100 and 200 nm groups, it was found that the contraction becomes more severe as the concentration of lumican increased (Fig. 7B, all *P* < 0.001). These results suggested that lumican treatment promoted collagen contraction in a dose‐dependent manner.

## Discussion

Lumican is a widely distributed small leucine‐rich repeat proteoglycan with diverse functions [28]. Several knockout mouse studies reveal that lumican is involved in the pathogenesis of fibrotic diseases [21, 22, 23]. Because joint contracture is also a kind of fibrotic disease, lumican may also have an effect on the pathogenesis of joint contracture. To test this hypothesis, this study aimed to investigate the role of lumican in the development of joint contracture. IHC showed that protein levels of lumican, α‐SMA, PCNA and TGF‐β were up‐regulated in the human fibrotic joint capsule as compared with normal tissues. *In vitro* cell culture study showed that exogenous lumican treatment (10–200 nm) significantly enhanced proliferation of synovial fibroblasts at 24 and 48 h in a dose‐dependent manner. Lumican treatment significantly promoted cell migration of synovial fibroblasts in a dose‐dependent manner. Immunofluorescence revealed that lumican treatment significantly up‐regulated α‐SMA proteins in synovial fibroblasts in a dose‐dependent manner. Real‐time RT‐PCR and western blot showed that lumican treatment significantly up‐regulated mRNA and protein levels of TGF‐β, MMP‐9, collagen and PAI‐1 in synovial fibroblasts in a dose‐dependent manner. However, lumican treatment did not affect the expression of SMAD3. Furthermore, the *in vitro* collagen lattice contraction assay showed that lumican treatment induced significant collagen lattice contraction at 48 h in a dose‐dependent manner. Taken together, these *in vitro* findings suggested that lumican promoted fibroblast–myofibroblast transition (myofibroblast activation) and collagen contracture, which was probably via activating TGF‐β1 signaling. To the best of our knowledge, this study reported the role of lumican in the pathogenesis of joint contracture for the first time.

Interestingly, lumican exhibits two completely opposite functions in the fibrotic diseases of different tissues or organs. In the study by Krishnan *et al*. [22] on hepatic fibrosis, lumican is up‐regulated in clinical tissue samples with hepatitis C virus infection. Lumican‐null mice have a significantly less severe extent of hepatic fibrosis and significantly higher protein level of α‐SMA and MMP‐13 [22]. These findings suggest a profibrotic role of lumican in the pathogenesis of hepatic fibrosis. Supporting this notion, our results also suggested a profibrotic function of lumican in the development of joint contracture. By contrast, lumican exhibits an antifibrotic function in the cornea. It is known that lumican maintains corneal transparency by inhibiting corneal collagen fibrillogenesis [16], promoting corneal epithelial wound healing [29, 30], regulating gene expression and maintaining corneal homeostasis [8]. Likewise, lumican‐null mice are susceptible to aging or isoproterenol‐induced myocardial fibrosis, suggesting that lumican plays an antifibrotic role in the pathogenesis of myocardial fibrosis [21]. The earlier‐mentioned findings suggest that lumican exerts opposite functions in the pathogenesis of varying fibrotic diseases. It is worth further elucidating the detailed molecular mechanism underlying the profibrotic and antifibrotic effects of lumican in these fibrotic diseases.

During pathological fibrosis, prolonged tissue injury or chronic inflammation can induce the proliferation of fibroblasts and myofibroblast activation [31]. Myofibroblast activation is a key event in the pathological fibrosis and tissue repair [32], in which myofibroblasts are the primary cells producing excessive ECM [31, 33]. Myofibroblasts have better contractile properties than fibroblasts [8], rendering these cells profibrotic property. It has been found that myofibroblasts are increased in the joint capsule after joint immobilization [7]. Hildebrand *et al*. [35] have reported that both myofibroblasts and α‐SMA (a myofibroblast marker) [34] protein are up‐regulated in the capsule tissue from human contractures elbows [35]. Consistently, their animal study reported that myofibroblast numbers and α‐SMA levels are also elevated in capsules from contractures knees of a rabbit model of joint contractures [36]. In this study, human fibrotic joint capsule tissues showed elevated levels of α‐SMA protein, which is in line with the observation from the animal model or clinical findings of joint contractures [25, 35, 36]. Nevertheless, our *in vitro* study further demonstrated that lumican‐treated synovial fibroblasts had elevated protein levels of α‐SMA, suggesting that exogenous lumican treatment can effectively induce myofibroblast activation from fibroblasts. Our results also showed that lumican‐treated synovial fibroblasts had increased levels of collagen. Increased production of ECM proteins (e.g. type I collagen) in the joint capsule is involved in the pathogenesis of joint contractures [37, 38].

In this study, we found that TGF‐β1 levels were elevated in human fibrotic joint capsule tissues. TGF‐β is an autocrine cytokine that can be produced by fibroblasts and then acts on fibroblasts to promote fibrotic responses [39], as well as increase the levels of myofibroblasts and α‐SMA [34, 40]. TGF‐β acts on fibroblasts and myofibroblasts to promote proliferation, migration, ECM production, fibrosis and myofibroblast activation [31, 41]. Our results showed that lumican treatment promoted proliferation, migration, α‐SMA expression and TGF‐β production in human synovial fibroblasts, suggesting that lumican treatment induced TGF‐β production by human synovial fibroblasts, which can stimulate proliferation, migration and myofibroblast activation in fibroblasts via an autocrine manner. Similarity, our IHC also found that PCNA protein was up‐regulated in the human fibrotic joint capsule, suggesting an increase in cell proliferation.

Our collagen lattice contraction assay showed that lumican treatment induced markedly collagen lattice contraction at 48 h in a dose‐dependent manner, suggesting that lumican can induce tissue contraction. The collagen lattice contraction assay is an *in vitro* model of tissue contraction with good reproducibility [42, 43]. Different from the animal model of joint contracture, the collagen lattice contraction assay can determine whether cell‐populated collagen hydrogels contract over time in a predictable and consistent manner [42].

Several limitations of this study should be pointed out. First, although we found that lumican and TGF‐β proteins were up‐regulated in human fibrotic joint capsule tissue and exogenous lumican‐treated synovial fibroblasts, we did not further elucidate the downstream molecules in TGF‐β signaling. It is known that lumican can stimulate cardiac fibrosis through increased TGF‐β production and phosphorylation of SMAD3 in cardiac fibroblasts [44]. Even though our data showed that lumican treatment did not affect the expression of SMAD3, we did not further determine the protein level of phosphorylated SMAD3. Thus, it remains unclear whether SMAD3 is a downstream molecule of lumican‐induced activation of TGF‐β signaling. In addition, although the *in vitro* collagen lattice contraction assay is a predictable and reliable method to evaluate tissue contraction, the *in vitro* observations of this study still need to be validated in an animal model of joint contracture. All of these limitations should be addressed in future studies.

In summary, our study suggested that lumican had a profibrotic function promoting the pathogenesis of joint contracture, which may activate the TGF‐β signaling pathway. These findings indicated that lumican might be a potential target for the treatment or prevention of joint contracture.

## Conflict of interest

The authors declare no conflict of interest.

## Author contributions

DX and TL contributed equally to this work. DS and KW conceived the study. TL drafted and wrote the manuscript. DX performed the experiments. ZZ contributed to the interpretation of data and review of the manuscript. RH performed the literature search. JR and SJ collected and assembled the data. LZ contributed to the language editing. All authors read and approved the final manuscript.

## Data Availability

The datasets generated and/or analyzed during the study are included within the article and are available from the corresponding authors on reasonable request.
